# Angiotensin-converting enzyme inhibitors vs. receptor blockers in heart failure with mildly reduced ejection fraction

**DOI:** 10.3389/fcvm.2026.1753965

**Published:** 2026-03-25

**Authors:** Kathrin Weidner, Michael Behnes, Marielen Reinhardt, Noah Abel, Alexander Schmitt, Felix Lau, Henning Johann Steffen, Svetlana Hetjens, Daniel Duerschmied, Ibrahim Akin, Tobias Schupp

**Affiliations:** 1 Department of Cardiology, Angiology, Haemostaseology and Medical Intensive Care, University Medical Centre Mannheim, Medical Faculty Mannheim, Heidelberg University, Mannheim, Germany; 2 Department of Medical Statistics and Biomathematics, Medical Faculty Mannheim, University of Heidelberg, Mannheim, Germany

**Keywords:** angiotensin receptor blockers, angiotensin-converting enzyme inhibitors, heart failure with mildly reduced ejection fraction, HFmrEF, pharmacotherapies

## Abstract

**Background:**

Evidence regarding the prognostic impact of angiotensin-converting enzyme inhibitors (ACEi) vs. receptor blockers (ARB) in heart failure with mildly reduced ejection fraction (HFmrEF) is limited.

**Methods:**

We retrospectively studied consecutive patients hospitalized with HFmrEF from 2016 until 2022 at a German university hospital. The prognostic impact of treatment with ACEi compared with ARB was investigated regarding the primary endpoint of all-cause mortality at 30 months. The key secondary endpoint was heart failure (HF)–related rehospitalization.

**Results:**

A total of 1,551 patients discharged on renin–angiotensin system inhibitors (ACEi: *n* = 1,055; ARB: *n* = 496) were included. Patients treated with ARB were older and had a higher burden of comorbidities. All-cause mortality at 30 months occurred in 251/1,055 (23.8%) patients treated with ACEi and in 147/496 (29.6%) patients treated with ARB [unadjusted hazard ratio [HR] = 0.762, 95% confidence interval [CI] 0.622–0.934; log-rank *p* = 0.009]. After multivariable adjustment, ACEi were still associated with improved long-term survival (adjusted HR = 0.786, 95% CI: 0.625–0.989; *p* = 0.040). This association was still found after propensity score matching (*n* = 440 per group) (23.2% vs. 29.5%; HR = 0.749, 95% CI: 0.578–0.971; *p* = 0.029). In contrast, the risk of HF-related rehospitalization at 30 months did not differ between the two groups in the unmatched cohort (12.7% vs. 14.5%; HR = 0.849, 95% CI: 0.638–1.130; *p* = 0.262) and after propensity score matching (16.1% vs. 14.8%; HR = 1.080, 95% CI: 0.772–1.512; *p* = 0.653).

**Conclusions:**

In patients hospitalized with HFmrEF, ACEi therapy was associated with reduced 30-month all-cause mortality compared with ARB, whereas the risk of HF-related rehospitalization did not differ.

## Introduction

Heart failure (HF) represents a major driver of morbidity and mortality worldwide and places an escalating burden on health systems (1). Although the mechanisms of HF with reduced ejection fraction (HFrEF) and preserved ejection fraction (HFpEF) are well characterized, HF with mildly reduced ejection fraction (HFmrEF) has more recently gained attention as a distinct clinical phenotype that warrants further investigation (2, 3). HFmrEF—defined by a left ventricular ejection fraction (LVEF) of 41%–49%—comprises a heterogeneous population that shares features with both HFrEF and HFpEF (2, 4). Despite growing recognition, evidence to guide therapy in HFmrEF remains limited, creating uncertainty for treatment decisions and guideline recommendations (2, 5).

Several drug classes—including β-blockers, angiotensin-converting enzyme inhibitors (ACEi)/receptor blockers (ARB), mineralocorticoid receptor antagonists (MRAs), and sodium–glucose cotransporter-2 (SGLT2) inhibitors—have proven benefits in HFrEF (2). In HFmrEF, treatment effects appear heterogeneous; notably, SGLT2 inhibitors have been shown to improve outcomes in both HFpEF and HFmrEF patients (6). Although ACEi/ARB treatment has been shown to improve outcomes in HFmrEF patients within registry-based studies (7), head-to-head comparisons of ARBs and ACEi are scarce. Prior studies comparing ACEi and ARB in patients with HFrEF or after myocardial infarction suggest either comparable outcomes (i.e., noninferiority of ARB to ACEi) or a potential advantage of ACEi in some post-MI populations (8–10). Data from HFmrEF patients diverge: irbesartan was neutral vs. placebo in I-PRESERVE, whereas *post-hoc* analyses of the CHARM programme (CHARM-Preserved) suggested that candesartan may reduce cardiovascular events vs. placebo (11, 12).

Therefore, this study investigates the comparative outcomes of HFmrEF patients treated with ACEi vs. ARB in a large retrospective cohort.

## Methods

### Study patients, design and data collection

For the present study, all consecutive patients hospitalized with HFmrEF at a University Medical Centre between January 2016 and December 2022 were included. Relevant clinical data pertaining to the index hospitalization were collected using the hospital's electronic information system. This included baseline characteristics, vital signs at admission, prior medical history, previous treatments, length of hospitalization and intensive care unit (ICU) stay, laboratory values, data from both non-invasive and invasive cardiac diagnostics, as well as device therapies. Specific data included echocardiographic findings, coronary angiography results, information on previously or newly implanted cardiac devices, and pharmacological treatments for HF at the time of discharge. All subsequent outpatient visits or rehospitalizations related to HF or adverse cardiac events were documented through the end of 2022. The study draws upon the “Heart Failure with Mildly Reduced Ejection Fraction Registry” (HARMER), a retrospective, single-center registry encompassing consecutive patients with HFmrEF hospitalized at the University Medical Center Mannheim (UMM), Germany (clinicaltrials.gov identifier: NCT05603390). The registry was conducted in accordance with the principles outlined in the Declaration of Helsinki and received approval from the Medical Ethics Committee II of the Medical Faculty Mannheim, University of Heidelberg, Germany (ethical approval code: 2022-818).

### Inclusion and exclusion criteria

All consecutive patients aged ≥18 years hospitalized with HFmrEF at a single institution were included. The diagnosis of HFmrEF was made according to the 2021 ESC Guidelines for the Diagnosis and Treatment of Acute and Chronic Heart Failure (2). Accordingly, all patients with a LVEF of 41%–49% and signs and/or symptoms of heart failure were included. Elevated levels of N-terminal prohormone of brain natriuretic peptide (NT-proBNP) and other evidence of structural heart disease were considered supportive. However, they were not mandatory for the diagnosis of HFmrEF. Transthoracic echocardiography was performed by cardiologists during routine clinical care, in accordance with current European guidelines (13). Echocardiographic operators were blinded to the final study analyses. For this study, all echocardiographic examinations and reports were reassessed *post hoc* by two independent cardiologists, with key echocardiographic measurements re-evaluated.

Ischemic cardiomyopathy was defined as the presence of significant coronary artery disease on coronary angiography or a documented history of myocardial infarction or coronary revascularization. Since risk stratification was based on the HF pharmacotherapy prescribed at the time of hospital discharge, patients who died during hospitalization were excluded. Furthermore, patients without treatment with either ACEi or ARB at index hospital discharge, as well as patients discharged on angiotensin receptor–neprilysin inhibitor (ARNI) or both ACEi plus ARB were excluded.

### Study endpoints

The primary endpoint was 30-month all-cause mortality. The key secondary endpoint was the risk of rehospitalization for worsening HF. HF-related hospitalization was defined as any readmission due to worsening HF requiring intravenous diuretic therapy. This included patients admitted primarily for HF exacerbation, as well as those admitted for other reasons but who either had worsening HF at admission or during the hospital stay.

### Statistical methods

Quantitative data were presented as medians with interquartile ranges (IQR) and were compared using the Student's *t*-test for normally distributed data or the Mann–Whitney *U*-test for nonparametric data, as appropriate. Deviations from a Gaussian distribution were assessed using the Kolmogorov–Smirnov test. Qualitative data were presented as absolute and relative frequencies and were compared using the chi-square test or Fisher's exact test, as appropriate.

Kaplan–Meier analyses were performed to compare patients treated with ACEi vs. ARB with regard to all-cause mortality and HF–related rehospitalization, and univariable hazard ratios (HRs) with 95% confidence intervals (CIs) were calculated. Furthermore, multivariable Cox proportional hazards regression models were applied to evaluate the independent prognostic impact of ACEi vs. ARB after adjustment for relevant clinical covariates. Results are presented as HRs with 95% CIs. Candidate variables for multivariable Cox regression included age, sex, ischemic cardiomyopathy, LVEF, chronic kidney disease, anemia, diabetes mellitus, atrial fibrillation, prior HF, and HF pharmacotherapies at discharge; variables were selected based on clinical relevance and prior literature. The proportional hazards assumption was assessed for each covariate using Schoenfeld residuals and the Supremum test. In case of a significant violation of the proportional hazards assumption, additional time-dependent analyses were performed by restricting follow-up to periods beyond 10 months to reassess model validity.

To account for potential confounding due to imbalances in baseline characteristics, propensity score matching was applied using a 1:1 nearest-neighbor matching approach without replacement. Propensity scores were derived from a multivariable logistic regression model including clinically relevant covariates known to affect prognosis in patients with HF.

Within the propensity score matched cohort, Kaplan–Meier analyses and univariable HRs were recalculated to assess the robustness of the association between ACEi vs. ARB therapy and clinical outcomes.

Results from all statistical tests were considered statistically significant for a two-sided *p* value ≤ 0.05. All statistical analyses were performed using SPSS (Version 28, IBM Corp., Armonk, NY, USA).

## Results

### Study population

From January 2016 to December 2022, 2,224 consecutive patients hospitalized with HFmrEF at our institution were screened for inclusion. Of the 2,184 patients included in the HARMER registry, 75 patients who died during index hospitalization, 555 patients without treatment with angiotensin-converting enzyme inhibitors (ACEi) or angiotensin receptor blockers (ARB) at discharge, as well as three patients receiving combined ACEi and ARB therapy were excluded for the present study.

The final study population comprised 1,551 patients discharged on renin–angiotensin system inhibitors, including 1,055 patients treated with ACEi and 496 patients treated with ARB (Table 1, **left column**). Among patients treated with ACE inhibitors at discharge, ramipril was the most frequently prescribed agent (*n* = 969; median 5 mg/d), followed by enalapril (*n* = 48; median 17.5 mg/d) and lisinopril (*n* = 32; median 20 mg/d). The most commonly prescribed ARB were candesartan (*n* = 333; median 16 mg/d), valsartan (*n* = 88; median 160 mg/d) and losartan (*n* = 21; median 100 mg/d). Patients treated with ARB were older [median 77 [IQR 69–84] vs. 74 [62–82] years; *p* = 0.001] and less frequently male (60.1% vs. 68.4%; *p* = 0.001). Furthermore, patients in the ARB group more often had a history of congestive HF, chronic kidney disease, and diabetes mellitus, whereas prior coronary artery disease and percutaneous coronary intervention were more common among patients treated with ACEi (Tables 1 and 2, **left column**).

**Table 1:** Baseline characteristics.

	Before propensity score matching					After propensity score matching				
Variables	ARB (*n* = 496)		ACEi (*n* = 1055)		p value	ARB (*n* = 440)		ACEi (*n* = 440)		p value
**Age**, median (IQR)	77	(69–84)	74	(62–82)	**0.001**	78	(70–84)	77	(68–84)	0.683
**Male sex**, n (%)	298	(60.1)	722	(68.4)	**0.001**	267	(60.7)	275	(62.5)	0.579
**Body mass index,** kg/m^2^, median (IQR)	27.5	(24.4–31.2)	26.8	(24.1–31.0)	0.105	27.5	(24.4–31.2)	27.2	(24.2–31.5)	0.918
**SBP**, mmHg, median (IQR)	144	(130–170)	145	(129–165)	0.571	144	(130–170)	146	(127–164)	0.631
**DBP**, mmHg, median (IQR)	80	(69–90)	80	(70–92)	0.375	80	(69–90)	80	(67–94)	0.733
**Heart rate**, bpm, median (IQR)	80	(66–93)	80	(69–95)	0.183	78	(65–90)	80	(67–94)	0.829
**Medical history**, *n* (%)										
Coronary artery disease	253	(51.0)	442	(41.9)	**0.001**	223	(50.7)	219	(49.8)	0.788
Prior myocardial infarction	140	(28.2)	270	(25.6)	0.273	122	(27.7)	130	(29.5)	0.551
Prior PCI	176	(35.5)	301	(28.5)	**0.006**	156	(35.5)	147	(33.4)	0.523
Prior CABG	56	(11.3)	110	(10.4)	0.608	53	(12.0)	61	(13.9)	0.422
Prior valvular surgery	21	(4.2)	39	(3.7)	0.609	17	(3.9)	16	(3.6)	0.859
Congestive heart failure	190	(38.3)	340	(32.2)	**0.019**	165	(37.5)	167	(38.0)	0.889
Decompensated heart failure < 12 months	64	(12.9)	111	(10.5)	0.167	55	(12.5)	52	(11.8)	0.757
Chronic kidney disease	183	(36.9)	278	(26.4)	**0.001**	166	(37.7)	141	(32.0)	0.077
Peripheral artery disease	66	(13.3)	112	(10.6)	0.121	60	(13.6)	52	(11.8)	0.419
Stroke	91	(18.3)	142	(13.5)	**0.012**	79	(18.0)	79	(18.0)	1.000
Liver cirrhosis	8	(1.6)	15	(1.4)	0.772	8	(1.8)	9	(2.0)	0.807
Malignancy	60	(12.1)	138	(13.1)	0.588	51	(11.6)	54	(12.3)	0.755
COPD	62	(12.5)	116	(11.0)	0.386	54	(12.3)	49	(11.1)	0.600
**Cardiovascular risk factors,** *n* (%)										
Arterial hypertension	464	(93.5)	817	(77.4)	**0.001**	417	(94.8)	414	(94.4)	0.659
Diabetes mellitus	221	(44.6)	381	(36.1)	**0.001**	199	(45.2)	180	(40.9)	0.196
Hyperlipidemia	187	(37.7)	326	(30.9)	**0.008**	167	(38.0)	167	(38.0)	1.000
Smoking										
Current	62	(12.5)	240	(22.7)	**0.001**	53	(12.0)	80	(18.2)	**0.011**
Former	112	(22.6)	166	(15.7)	**0.001**	97	(22.0)	72	(16.4)	**0.032**
Family history	41	(8.3)	127	(12.0)	**0.026**	36	(8.2)	45	(10.2)	0.294
**Comorbidities at index hospitalization**, *n* (%)										
Acute coronary syndrome										
Unstable angina	31	(6.3)	55	(5.2)	0.405	29	(6.6)	31	(7.0)	0.789
STEMI	31	(6.3)	123	(11.7)	**0.001**	31	(7.0)	30	(6.8)	0.894
NSTEMI	58	(11.7)	170	(16.1)	**0.022**	52	(11.8)	61	(13.9)	0.365
Acute decompensated heart failure	119	(24.0)	212	(20.1)	0.081	108	(24.5)	102	(23.2)	0.635
Cardiogenic shock	9	(1.8)	26	(2.5)	0.421	9	(2.0)	8	(1.8)	0.807
Atrial fibrillation	214	(43.1)	398	(37.7)	**0.042**	183	(41.6)	186	(42.3)	0.838
Cardiopulmonary resuscitation	6	(1.2)	26	(2.5)	0.105	6	(1.4)	6	(1.4)	1.000
Out-of-hospital	2	(0.4)	15	(1.4)	0.072	2	(0.5)	3	(0.7)	0.654
In-hospital	4	(0.8)	11	(1.0)	0.658	4	(0.9)	3	(0.7)	0.704
Stroke	61	(12.3)	123	(11.7)	0.716	57	(13.0)	53	(12.0)	0.683

ACEi, angiotensin-converting-enzyme inhibitor; ARB, angiotensin receptor blocker; ARNI, angiotensin receptor neprilysin inhibitor; ASA, acetylsalicylic acid; CABG, coronary artery bypass grafting; COPD, chronic obstructive pulmonary disease; DBP, diastolic blood pressure; DOAC, directly acting oral anticoagulant; ICD, implantable cardioverter defibrillator; IQR, interquartile range; (N)STEMI, non-ST-segment elevation myocardial infarction; SBP, systolic blood pressure; SGLT2, sodium glucose linked transporter 2.

Level of significance *p* ≤ 0.05. Bold type indicates statistical significance.

### Prognostic impact of ACEi vs. ARB treatment before propensity score matching

**Figure 1:**
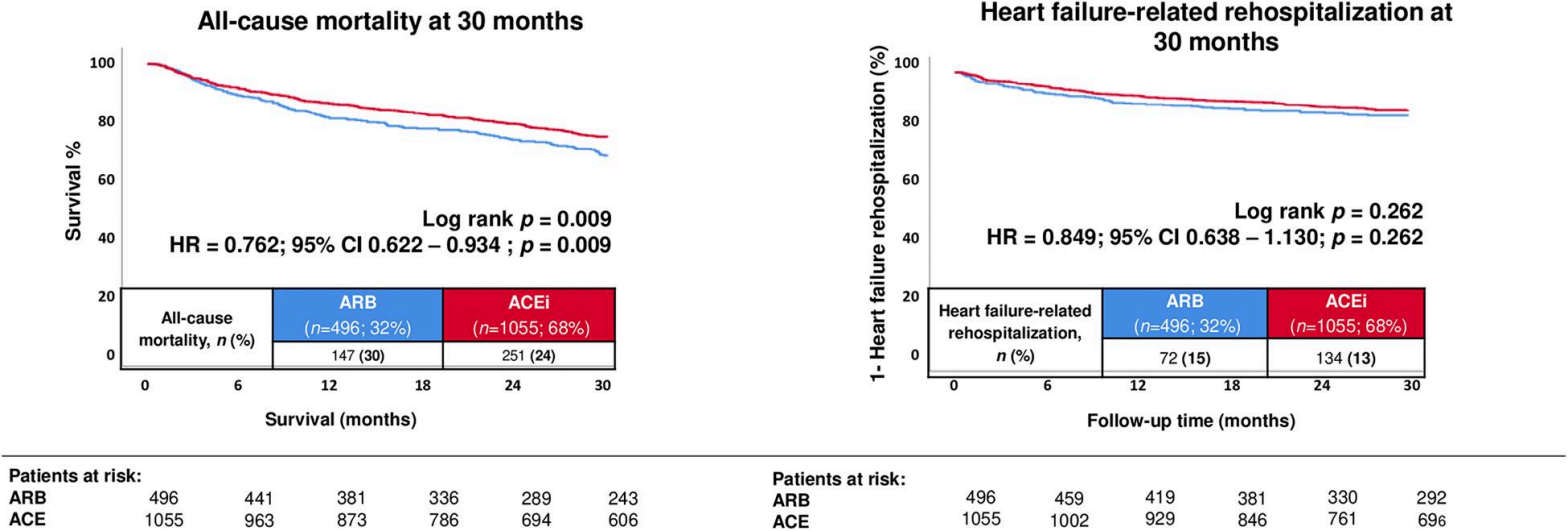
Prognostic impact of treatment with angiotensin-converting enzyme inhibitors (ACEi) and angiotensin receptor blockers (ARB) on the risk of all-cause mortality at 30 months (**left panel**), as well as on the risk of heart failure–related rehospitalization (**right panel**) in the unmatched cohort.

In the unmatched cohort, the primary endpoint of all-cause mortality occurred in 23.8% of patients treated with ACEi and in 29.6% of patients treated with ARB during a median follow-up of 30 months. In Kaplan–Meier analysis, treatment with ACEi was associated with a significantly lower risk of all-cause mortality compared with ARB (HR: 0.762, 95% CI: 0.622–0.934; log-rank *p* = 0.009) (Figure 1; Table 3, **left panel**). With regard to the key secondary endpoint, HF–related rehospitalization at 30 months occurred in 12.7% of ACEi-treated patients and in 14.5% of ARB-treated patients, with no significant difference between both groups (HR: 0.849, 95% CI: 0.638–1.130; *p* = 0.262) (Figure 1; Table 3, **left column**).

**Table 2:** Heart failure-related and procedural data.

	Before propensity score matching				After propensity score matching					
Variables	ARB (*n* = 496)		ACEi (*n* = 1055)		p value	ARB (*n* = 440)		ACEi (*n* = 440)		p value
**Heart failure etiology**, *n* (%)										
Ischemic cardiomyopathy	325	(65.5)	683	(64.7)	**0.015**	291	(66.1)	293	(66.6)	0.449
Non-ischemic cardiomyopathy	35	(7.1)	63	(6.0)		31	(7.0)	25	(5.7)	
Hypertensive cardiomyopathy	55	(11.1)	72	(6.8)		50	(11.4)	39	(8.9)	
Congenital heart disease	0	(0.0)	1	(0.1)		0	(0.0)	0	(0.0)	
Valvular heart disease	13	(2.6)	38	(3.6)		9	(2.0)	14	(3.7)	
Tachycardia-associated	15	(3.0)	40	(3.8)		12	(2.7)	16	(3.6)	
Tachymyopathy	7	(1.4)	16	(1.5)		7	(1.6)	7	(1.6)	
Pacemaker-induced cardiomyopathy	6	(1.2)	6	(0.6)		5	(1.1)	2	(0.5)	
Unknown	40	(8.1)	136	(12.9)		35	(8.0)	44	(10.0)	
**NYHA functional class,** *n* (%)										
I/II	349	(70.4)	762	(72.2)	0.072	306	(69.5)	310	(70.4)	0.351
III	98	(19.8)	212	(20.1)		91	(20.7)	86	(19.5)	
IV	49	(9.9)	81	(7.7)		43	(9.8)	44	(10.0)	
**Echocardiographic data**										
LVEF, %, median (IQR)	45 (45–47)		45 (45–47)		0.212	45 (45–46)		45 (45–46)		0.129
IVSd, median (IQR)	12 (11–13)		12 (11–13)		0.075	13 (12–14)		12 (12–13)		0.948
LVEDD, mm, median (IQR)	49 (45–53)		49 (44–54)		**0.025**	49 (48–55)		54 (49–59)		0.601
TAPSE, mm, median (IQR)	20 (18–23)		20 (18–23)		0.825	20 (20–22)		21 (20–22)		0.284
Diastolic dysfcunction, *n* (%)	380	(76.6)	759	(71.9)	0.052	332	(75.5)	331	(75.2)	0.938
Moderate-severe aortic stenosis, *n* (%)	46	(9.3)	96	(9.1)	0.911	40	(9.1)	53	(12.0)	0.154
Moderate-severe aortic regurgitation, *n* (%)	19	(3.8)	36	(3.4)	0.678	18	(4.1)	18	(4.1)	1.000
Moderate-severe mitral regurgitation, *n* (%)	61	(12.3)	111	(10.5)	0.299	53	(12.0)	54	(12.3)	0.918
Moderate-severe tricuspid regurgitation, *n* (%)	76	(15.3)	135	(12.8)	0.176	66	(15.0)	79	(18.0)	0.238
VCI, median (IQR)	20 (17–26)		19 (15–25)		0.120	21 (19–23)		20 (18–22)		0.579
Aortic root, mm, median (IQR)	32 (29–36)		33 (30–36)		0.360	33 (32–33)		33 (32–33)		0.638
**Coronary angiography,** *n* (%)	220	(44.4)	540	(51.2)	**0.012**	197	(45)	227	(52)	**0.043**
No evidence of coronary artery disease	41	(18.6)	91	(16.9)	0.725	35	(18)	41	(18)	0.386
1-vessel disease	45	(20.5)	102	(18.9)		40	(20)	33	(15)	
2-vessel disease	43	(19.5)	124	(23.0)		39	(20)	47	(21)	
3-vessel disease	91	(41.4)	223	(41.3)		83	(42)	106	(47)	
Chronic total occlusion	23	(10.5)	70	(13.0)	0.339	22	(11.2)	26	(11.5)	0.525
PCI, n (%)	110	(50.0)	314	(58.1)	**0.040**	101	(51.3)	113	(49.8)	0417
Sent to CABG, n (%)	14	(6.4)	29	(5.4)	0.591	13	(6.6)	10	(4.4)	0.218
**Baseline laboratory values**, median (IQR)										
Potassium, mmol/L	3.9 (3.6–4.2)		3.9 (3.6–4.2)		0.914	3.9 (3.8–3.9)		3.8 (3.8–3.9)		0.719
Sodium, mmol/L	139 (137–141)		139 (137–141)		0.579	139 (138–139)		139 (139–139)		0.491
Creatinine, mg/dL	1.13 (0.92–1.52)		1.03 (0.84–1.33)		**0.001**	1.5 (1.4–1.6)		1.3 (1.3–1.5)		0.105
eGFR, mL/min/1.73 m^2^	58.4 (42.2–78.6)		69.5 (50.2–89.7)		**0.001**	60.2 (57.77–62.8)		62.8 (60.5–65.1)		0.088
Hemoglobin, g/dL	12.4 (10.5–13.9)		12.6 (10.7–14.2)		0.161	12.2 (12.0–12.4)		12.3 (12.0–12.5)		0.689
WBC count, x 10^9^/L	7.99 (6.42–9.79)		8.25 (6.47–10.21)		0.201	8.5 (8.1–8.9)		9.3 (8.3–10.2)		0.517
Platelet count, x 10^9^/L	222 (175–282)		230 (186–294)		**0.011**	235 (226–244)		247 (238–256)		**0.044**
HbA1c, %	6.1 (5.6–7.1)		5.9 (5.5–6.7)		**0.002**	6.4 (6.2–6.5)		6.3 (6.1–6.4)		0.186
LDL- cholesterol, mg/dL	91 (70–120)		101 (77–129)		**0.001**	95 (91–100)		103 (98–108)		**0.035**
HDL- cholesterol, mg/dL	43 (35–52)		41 (34–50)		**0.049**	45 (43–47)		44 (42–45)		0.483
C-reactive protein, mg/L	10.1 (2.9–37.2)		12.0 (3.2–40.0)		0.112	29 (25–33)		29 (25–33)		0.371
NT-pro BNP, pg/mL	2598 (1037–6383)		2290 (1003–5401)		0.192	8611 (5394–11828)		7333 (4486–10181)		0.811
NT-pro BNP eGFR corrected, pg/mL	1544 (625–3235)		1540 (682–3547)		0.706	3455 (2407–4503)		2654 (1995–3311)		0.927
Cardiac troponin I, µg/L	0.02 (0.02–0.14)		0.04 (0.02–0.33)		**0.001**	0.02 (0.02–0.14)		0.03 (0.02–0.18)		0.305
**Medication at discharge**, *n* (%)										
ACEi	0	(0.0)	1055	(100.0)	**0.001**	0	(0.0)	440	(100)	**0.001**
ARB	496	(100.0)	0	(0.0)	**0.001**	440	(100)	0	(0.0)	**0.001**
Beta-blocker	403	(81.3)	882	(83.6)	0.252	354	(80.5)	364	(82.7)	0.217
Aldosterone antagonist	82	(16.5)	154	(14.6)	0.322	76	(17.3)	59	(13.4)	0.067
SGLT2-inhibitor	29	(5.8)	40	(3.8)	0.067	28	(6.8)	24	(5.4)	0.334
Loop diuretics	269	(54.2)	486	(46.1)	**0.003**	238	(54.1)	227	(51.6)	0.250
Statin	379	(76.4)	781	(74.0)	0.313	336	(76.4)	339	(77.0)	0.437
Digitalis	26	(5.2)	56	(5.3)	0.957	21	(4.8)	27	(6.1)	0.229
Amiodarone	11	(2.2)	26	(2.5)	0.766	9	(2.0)	12	(2.7)	0.330
ASA	265	(53.4)	595	(56.4)	0.272	242	(55.0)	245	(55.7)	0.446
P2Y12-inhibitor	176	(35.5)	409	(38.8)	0.213	160	(36.4)	158	(35.9)	0.472
DOAC	187	(37.7)	334	(31.7)	**0.019**	159	(36.1)	157	(35.7)	0.472
Vitamin k antagonist	36	(7.3)	70	(6.6)	0.650	30	(6.8)	29	(6.6)	0.500

ACEi, angiotensin-converting enzyme inhibitor; ARB, angiotensin receptor blocker; ASA, acetylsalicylic acid; AV Vmax, aortic valve maximal velocity; CABG, coronary artery bypass grafting; DOAC, directly acting oral anticoagulant; eGFR, estimated glomerular filtration rate; HbA1c, glycated hemoglobin; HDL, high-density lipoprotein; IQR, interquartile range; IVSd, Interventricular septal end diastole; LA, left atrial; LDL, low-density lipoprotein; LVEDD, Left ventricular end-diastolic diameter; LVEF, left ventricular ejection fraction; MCH, mean corpuscular hemoglobin; MCHC, mean corpuscular hemoglobin concentration; MCV, mean corpuscular volume; NT-pro BNP, aminoterminal pro-B-type natriuretic peptide; NYHA, New York Heart Association; PCI, percutaneous coronary intervention; SGLT2, sodium glucose linked transporter 2; TAPSE, tricuspid annular plane systolic excursion; TRPG max, maximal tricuspid regurgitation pressure gradient; TR Vmax, maximal tricuspid regurgitation velocity; VCI, Vena cava inferior; WBC, white blood cells.

Level of significance *p* ≤ 0.05. Bold type indicates statistical significance.

### Dose-dependent analysis

**Figure 2:**
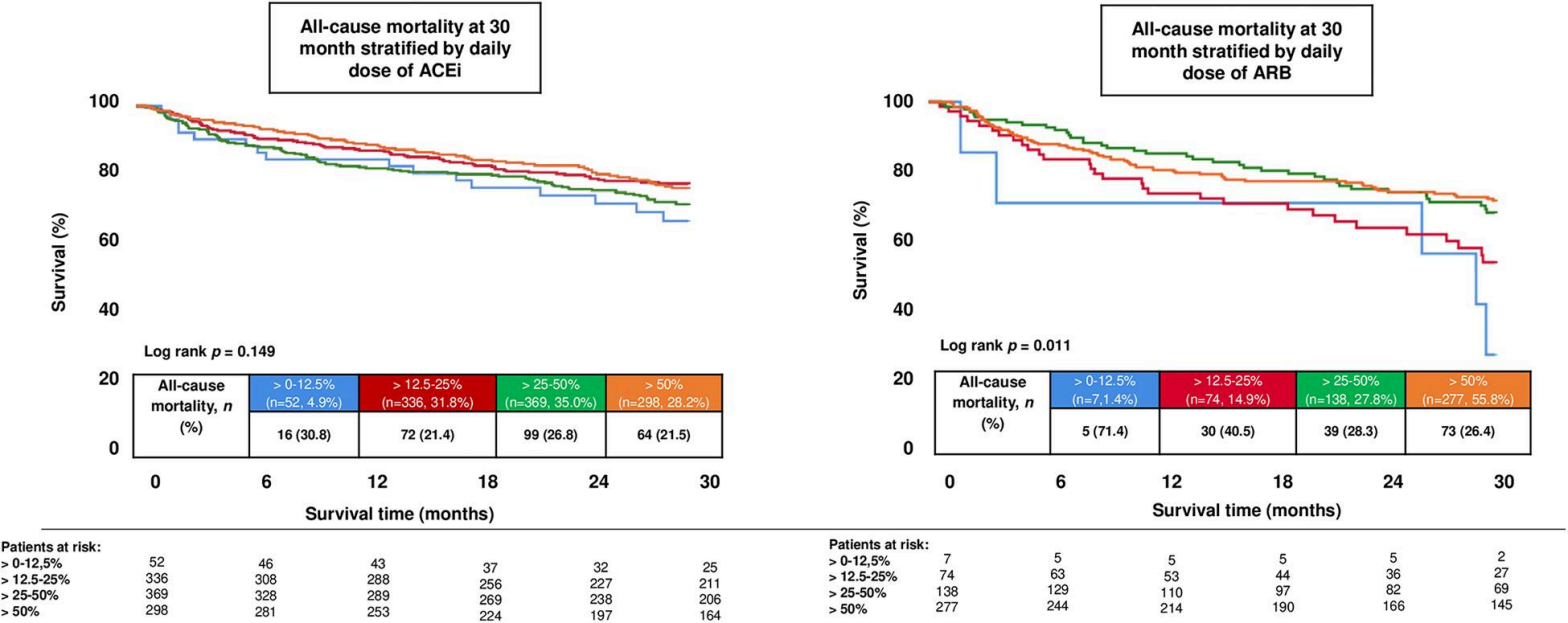
Dose-dependent association between treatment with angiotensin-converting enzyme inhibitors (ACEi) and angiotensin receptor blockers (ARB) and long-term outcomes. Kaplan–Meier curves display all-cause mortality at 30 months stratified by discharge dose categories normalized to guideline-recommended target doses (0%–12.5%, 12.5%–25%, 25%–50%, and >50%) for ACEi (**left panel**) and ARB (**right panel**).

In a dose-dependent analysis, patients were stratified according to the prescribed daily dose of renin–angiotensin system inhibitors at discharge. In patients treated with ACEi, higher dose levels were associated with a significantly improved long-term survival (log-rank *p* = 0.011) (Figure 2, **left panel**). In contrast, no significant dose–response relationship was observed in patients treated with ARB (log-rank *p* = 0.149). It should be noted that the observed differences may be influenced by the limited number of patients and events, which restricts statistical power; therefore, these findings should be interpreted cautiously (Figure 2, **right panel**).

### Multivariable cox regression

In multivariable Cox regression analysis, treatment with ACEi remained independently associated with a lower risk of all-cause mortality at 30 months compared with ARB (adjusted HR: 0.786, 95% CI: 0.625–0.989; *p* = 0.040), whereas no significant association was observed for HF–related rehospitalization (adjusted HR: 1.059, 95% CI: 0.776–1.446; *p* = 0.716) (Table 4). Assessment of Schoenfeld residuals demonstrated that the proportional hazards assumption was met for most covariates. For selected variables, the assumption was violated in the overall follow-up period (Supremum test *p* < 0.05). However, when restricting the analysis to follow-up beyond 10 months, these violations were no longer present (all *p* > 0.05), indicating that the proportional hazards assumption was met in the time-restricted models.

**Table 3:** Follow-up data, primary and secondary endpoints.

	Before propensity score matching							After propensity score matching						
Variables	ARB (*n* = 496)		ACEi (*n* = 1055)		HR	95% CI	p value	ARB (*n* = 440)		ACEi (*n* = 440)		HR	95% CI	p value
**Primary endpoints**, *n* (%)														
All-cause mortality, at 30 months	147	(29.6)	251	(23.8)	0.762	0.622–0.934	**0.009**	130	(29.5)	102	(23.2)	0.749	0.578–0.971	**0.029**
**Secondary endpoints**, *n* (%)														
All-cause mortality, at 12 months	94	(19.0)	148	(14.0)	0.725	0.560–0.938	**0.015**	87	(19.8)	66	(15.0)	0.742	0.539–1.022	0.068
Heart failure related rehospitalization, at 12 months	55	(11.1)	87	(8.2)	0.730	0.521–1.023	0.068	50	(11.4)	48	(10.9)	0.953	0.641–1.416	0.811
Heart failure related rehospitalization, at 30 months	72	(14.5)	134	(12.7)	0.849	0.638–1.130	0.262	65	(14.8)	71	(16.1)	1.080	0.772–1.512	0.653
Cardiac rehospitalization, at 30 months	115	(23.2)	245	(23.2)	0.981	0.786–1.224	0.862	103	(23.4)	116	(26.4)	1.125	0.863–1.647	0.385
Coronary revascularization, at 30 months	32	(6.5)	89	(8.4)	1.306	0.872–1.956	0.195	31	(7.0)	35	(8.0)	1.122	0.692–1.820	0.639
Acute myocardial infarction, at 30 months	21	(4.2)	30	(2.8)	0.655	0.375–1.145	0.137	20	(4.5)	14	(3.2)	0.685	0.346–1.356	0.277
Stroke, at 30 months	19	(3.8)	26	(2.5)	0.630	0.348–1.137	0.125	18	(4.1)	8	(1.8)	0.435	0.189–1.000	**0.050**
MACCE, at 30 months	181	(36.5)	354	(33.6)	0.891	0.745–1.066	0.207	164	(37.3)	142	(32.3)	0.835	0.667–1.045	0.115
**Follow-up data**, median (IQR)														
Hospitalization time	8 (5–14)		8 (5–14)		-	-	0.977	8 (5–14)		8 (6–14)		-	-	0.522
ICU time	0 (0–1)		0 (0–1)		-	-	0.060	0 (0–1)		0 (0–1)		-	-	0.524
Follow-up time	886 (393–1538)		1060 (530–1788)		-	-	**0.001**	879 (369–1,508)		1034 (505–1,761)		-	-	**0.011**

ACEi, angiotensin-converting enzyme inhibitor; ARB, angiotensin receptor blocker; CI, confidence interval; HR, hazard ratio; ICU, intensive care unit; MACCE, major adverse cardiac and cerebrovascular events.

Level of significance *p* ≤ 0.05. Bold type indicates statistical significance.

Subgroup analyses were performed using multivariable Cox regression models adjusting for clinically relevant covariates. In these prespecified subgroup analyses, treatment with ACE inhibitors was associated with a lower risk of 30-month all-cause mortality compared with ARB in several patient subgroups. Specifically, ACEi therapy was associated with improved survival in patients aged >75 years, those with chronic kidney disease, patients with LVEF ≥45%, patients without atrial fibrillation, and patients with ischemic cardiomyopathy. In contrast, no significant association between ACEi and all-cause mortality was observed in younger patients, those without chronic kidney disease, patients with LVEF <45%, atrial fibrillation, or non-ischemic cardiomyopathy (Table 5).

**Table 4:** Multivariate Cox regression analyses with regard to all-cause mortality and heart failure related rehospitalization at 30 months.

	All-cause mortality			Heart failure related rehospitalization		
Variables	HR	95% CI	p value	HR	95% CI	p value
Age (per year increase)	1.038	1.026–1.050	**0.001**	1.016	1.001–1.031	**0.043**
Male sex	1.392	1.097–1.767	**0.007**	0.793	0.584–1.077	0.138
BMI (per kg/m^2^ increase)	0.958	0.935–0.983	**0.001**	1.034	1.004–1.064	**0.025**
Prior congestive HF	1.079	0.857–1.357	0.519	1.866	1.368–2.545	**0.001**
CKD	1.493	1.172–1.901	**0.001**	1.555	1.116–2.166	**0.009**
Arterial hypertension	0.811	0.590–1.115	0.197	0.967	0.606–1.543	0.887
Diabetes mellitus	1.086	0.860–1.372	0.487	1.191	0.870–1.632	0.275
Malignancy	2.892	2.252–3.714	0.001	0.916	0.578–1.452	0.708
Ischemic cardiomyopathy	0.744	0.595–0.932	0.010	1.121	0.813–1.545	0.485
TAPSE <17 mm	1.233	0.944–1.612	0.124	1.039	0.727–1.486	0.834
Acute decompensated HF	1.508	1.184–1.920	**0.001**	2.443	1.785–3.343	**0.001**
Moderate/severe aortic stenosis	1.352	0.992–1.842	0.056	1.504	0.999–2.265	0.051
Anemia	1.757	1.370–2.254	**0.001**	1.306	0.941–1.813	0.111
ACEi vs. ARB	0.786	0.625–0.989	**0.040**	1.059	0.776–1.446	0.716

ACE, angiotensin converting enzyme inhibitor; ARB, angiotensin receptor blocker; BMI, body mass index; CI, confidence interval; CKD, chronic kidney disease; HF, heart failure; HR, hazard ratio; NYHA, New York Heart Association; TAPSE, tricuspid annular plane systolic excursion.

Level of significance *p* ≤ 0.05. Bold type indicates statistical significance.

### Prognostic impact of ACEi vs. ARB treatment after propensity score matching

To account for potential confounding due to the heterogeneous distribution of baseline characteristics, propensity score matching was applied in a 1:1 ratio. Based on predefined clinical variables, 440 patients treated with ARB were successfully matched with 440 patients treated with ACEi, resulting in a propensity score matched cohort of 880 patients (Tables 1 and 2, **right column**). After propensity score matching, baseline characteristics were well balanced between both groups with no significant differences regarding age [median 78 (70–84) vs. 77 (68–84) years; *p* = 0.683], sex (60.7% vs. 62.5% male; *p* = 0.579), cardiovascular risk factors, major comorbidities, or prior cardiac history (Table 1, **right column**). After propensity score matching, HF etiology, echocardiographic parameters and procedural characteristics were well balanced between both groups, with ischemic cardiomyopathy remaining the leading cause of HF (66.1% in the ARB group vs. 66.6% in the ACEi group; *p* = 0.449) and a comparable median LVEF [45% (45–46) vs. 45% (45–46); *p* = 0.129] (Table 2, **right column**).

**Table 5:** Prognostic impact of ACEi vs. ARB within pre-specified subgroups after multivariable adjustment with regard to all-cause mortality at 30 months.

Variables	HR	95% CI	p value
Age > 75	0.759	0.579–0.996	**0.047**
Age ≤ 75	0.743	0.471–1.145	0.173
Chronic kidney disease	0.664	0.482–0.914	**0.012**
No chronic kidney disease	0.998	0.709–1.403	0.989
LVEF < 45%	0.879	0.662–1.169	0.376
LVEF ≥ 45%	0.552	0.361–0.846	**0.006**
Atrial fibrillation	0.950	0.684–1.319	0.759
No atrial fibrillation	0.625	0.450–0.866	**0.005**
Ischemic cardiomyopathy	0.546	0.406–0.733	**0.001**
No ischemic cardiomyopathy	1.278	0.853–1.915	0.233

CI, confidence interval; CKD, chronic kidney disease; HF, heart failure; HR, hazard ratio; LVEF, left ventricular ejection fraction.

Level of significance *p* ≤ 0.05. Bold type indicates statistical significance.

Multivariable Cox regression models were adjusted for age, sex, BMI, body mass index; CKD, chronic kidney disease; ADHF, acute decompensated heart failure; anemia, and ischemic cardiomyopathy.

**Figure 3:**
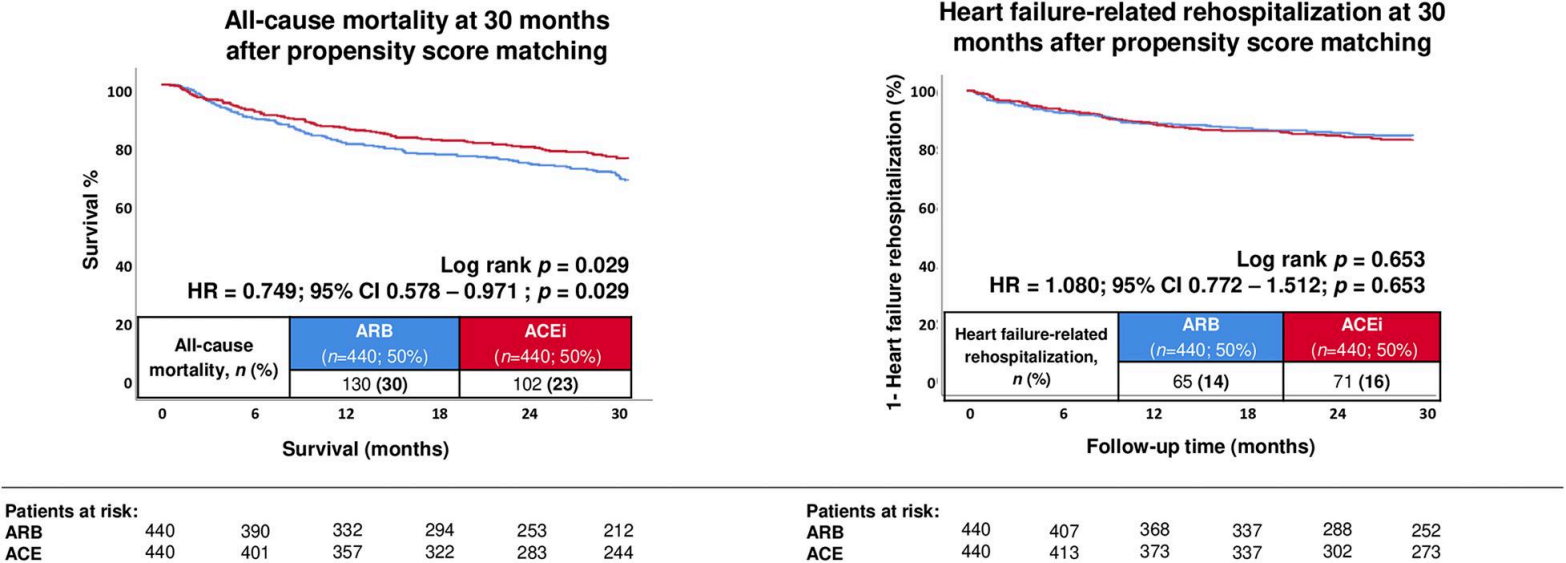
Prognostic impact of treatment with angiotensin-converting enzyme inhibitors (ACEi) and angiotensin receptor blockers (ARB) on the risk of all-cause mortality at 30 months (**left panel**), as well as on the risk of HF–related rehospitalization (**right panel**) after propensity score matching.

In the propensity score matched cohort, the prognostic advantage of ACEi regarding all-cause mortality remained consistent. All-cause mortality at 30 months occurred in 23.2% of ACEi-treated patients and in 29.5% of ARB-treated patients (HR: 0.749, 95% CI: 0.578–0.971; log-rank *p* = 0.029) (Figure 3; Table 3, **right column**). In contrast, HF–related rehospitalization at 30 months did not differ significantly between both groups after matching (16.1% in the ACEi group vs. 14.8% in the ARB group; HR: 1.080, 95% CI: 0.772–1.512; *p* = 0.653) (Figure 3; Table 3, **right column**).

## Discussion

In this single-center cohort of patients with HFmrEF, treatment with ACEi was associated with lower risk of long-term all-cause mortality as compared to ARB, which remained statistically significant after multivariable adjustment and propensity score matching. Secondary outcomes, specifically the risk of HF-related rehospitalization and other cardiovascular events did not differ significantly between the two groups. The benefit of ACEi compared with ARB appeared more pronounced in patients aged >75 years, those with chronic kidney disease, patients with LVEF ≥45%, individuals without atrial fibrillation, and patients with ischemic cardiomyopathy. These findings underscore the heterogeneity of HFmrEF and suggest that the prognostic impact of renin–angiotensin system inhibition may vary by underlying phenotype. Given the observational design, subgroup results should be interpreted cautiously and considered hypothesis-generating.

Evidence regarding the prognostic impact of ACEi vs. ARB is heterogeneous and derives largely from HFrEF trial populations, with additional insights mainly from subgroup analyses in post–myocardial infarction and rhythmology cohorts, rather than from studies specifically designed for HFmrEF (8–11, 14–16). Consistent with earlier trials, ARB demonstrated no superiority over ACEi in post-MI patients with HF and/or left ventricular dysfunction, supporting the applicability of this evidence to HFmrEF, a predominantly ischemic phenotype (9, 10).

In our study, guideline-directed therapies at discharge (β-blockers, mineralocorticoid receptor antagonists, early SGLT2i uptake) did not differ meaningfully, limiting confounding from other life-prolonging agents; PCI use during the index hospitalization was slightly higher with ACEi but was accounted for in the multivariable model for HF etiology and ischemic cardiomyopathy. In a ventricular arrhythmia cohort, ACEi and ARB showed similar three-year mortality and arrhythmic events; an apparent reduction in cardiac rehospitalizations with ACEi was no longer demonstrated after propensity matching (17). Differences in population (secondary prevention with more ICD carriers), endpoint definitions, and follow-up likely explain discrepancies vs. HF cohorts (17). In LVEF ranges overlapping HFmrEF, ARB effects are still inconsistent: irbesartan was neutral vs. placebo, whereas *post-hoc* analyses suggest candesartan may reduce cardiovascular events (11, 18). Observational studies also disagree—one registry favored candesartan over losartan, while a nationwide cohort found no overall difference and highlighted dose dependence, with high-dose losartan approximating candesartan (14, 19). Overall, the evidence remains inconclusive, underscoring the need for HFmrEF-specific analyses.

Physiological considerations provide a mechanistic rationale for a potential mortality signal with ACEi: reducing angiotensin II while increasing bradykinin enhances endothelial B2-receptor signaling with downstream nitric-oxide release, vasodilation, and anti-hypertrophic/anti-fibrotic effects (20). A potential ACE–B2 receptor “cross-talk” may also counter B2-receptor desensitization and internalization—effects not shared by ARB; moreover, kinin potentiation under ACE inhibition can occur rapidly and is not fully reproduced by blocking other kininases, suggesting a bradykinin-mediated component beyond simple Renin–angiotensin–aldosterone system (RAAS) suppression (20).

Across renin–angiotensin strategies, angiotensin receptor–neprilysin inhibition (ARNI; sacubitril/valsartan) was superior to ACE inhibition in reduced LVEF, lowering cardiovascular death and HF-related hospitalizations as compared to enalapril (21). In LVEF ranges overlapping HFmrEF, ARNI did not significantly outperform valsartan overall, with signals toward greater benefit at the lower EF spectrum nearer HFrEF (22). After acute myocardial infarction however, ARNI did not reduce the composite of cardiovascular death or incident HF vs. ramipril (10). In line, the prescription rates of ARNI were rather low in the present cohort, which warrants further consideration.

Taken together with head-to-head data from neighboring populations showing no superiority of ARB over ACEi (8–10), and observational evidence suggesting molecule- and dose-dependence within the ARB class (14, 19), the choice of RAAS blockade in HFmrEF should be embedded within contemporary background therapy including SGLT2i across HFmrEF/HFpEF and selective use of MRAs and β-blockers by phenotype (2, 6).

Overall, our findings suggest that ACEi may confer a survival advantage over ARB in HFmrEF, a finding that warrants confirmation in prospective randomized trials.

## Study limitations

The study has several limitations. Due to the retrospective and single-centre study design, results may be influenced by measured and unmeasured confounding despite multivariable adjustment and propensity score matching; therefore, causal inferences cannot be drawn. Related to the registry-based study design, new prescriptions, discontinuation, up- and down-titration of pharmacotherapies were not available for the present study. Thus, analyses are based on discharge medication only, and we cannot account for treatment modifications during follow-up (including persistence, adherence, and switching between ACEi and ARB). Furthermore, side effects of HF-related pharmacotherapies were not assessed within the present registry and drug intolerance was not systematically captured. Although discharge dose information was available for a subset and exploratory dose-group analyses were performed, long-term dose exposure and target dose achievement could not be assessed.

HF-related rehospitalizations were assessed at our institution only, as no cross-institutional claims/registry data were available, which may have led to under-ascertainment and potentially attenuated between-group differences. The proportion of patients with HFmrEF treated with ARNI was rather low during the study period and could not be evaluated, which may limit generalizability to contemporary settings with higher ARNI penetration. Finally, causes of death beyond during index hospitalization were not available for the present study; therefore, cardiovascular vs. non-cardiovascular mortality could not be analyzed. The single-centre German tertiary-care setting may further limit generalizability to other healthcare systems and more diverse populations.

## Conclusions

In this real-world HFmrEF registry, ACEi therapy was consistently associated with lower 30-month all-cause mortality compared with ARB.

## Data Availability

The original contributions presented in the study are included in the article/Supplementary Material, further inquiries can be directed to the corresponding author.
